# ESR1 and ESR2 genotypes and the age at menarche in idiopathic scoliosis

**DOI:** 10.1186/1748-7161-9-S1-P8

**Published:** 2014-12-04

**Authors:** Piotr Janusz, Tomasz Kotwicki, Miroslaw Andrusiewicz, Malgorzata Kotwicka, Dariusz Czaprowski, Mateusz Kozinoga

**Affiliations:** 1 Spine Disorders Unit, Department of Pediatric Orthopedics and Traumatology, University of Medical Sciences, Poznan, Poland; 2 Department of Cell Biology University of Medical Sciences, Poznan, Poland; 3 Department of Physiotherapy, Józef Rusiecki University College, Olsztyn, Poland

## Background

Environmental and genetic factors have influence on the age at menarche (AAM). Disturbance of the AAM in patients with idiopathic scoliosis (IS) were postulated [1]. Estrogen receptor genes 1 and 2 (ESR1, ESR2) single nucleotide polymorphisms (SNP) in IS were suggested to have some association with predisposition to IS [2]. ESR SNPs were reported to have association with AAM in healthily females [3].

## Aim

The purpose of the study was to investigate associations of the ESR1 and ESR2 SNPs with AAM in IS patients.

## Material

227 Caucasian females from Central Europe (Poland) with idiopathic scoliosis were included into this trial. The AAM in months was established in each case. Four SNPs were investigated with use of the restriction enzymes: in ESR1 rs9340799 and rs2234693 with XbaI and PvuII enzymes, in ESR2 rs4986938 and rs1256049 with Alu and RasI enzymes respectively. The statistic calculation was done with ANOVA, t-Student, or Kruskal-Wallis tests. P value 0.05 was considered as significant. The mean and SD values are presented in months.

## Results

All genotypes followed Hardy-Weinberg Equilibrium. The mean AAM for all patients was 154.7±14.3.

Genotypes distribution, mean and SD AAM values were:

XbaI - AA (N=76, 153.8±13.1), AG (N=113, 155.6±16.3) GG (N=38, 154.2±13.0) p=0.7613;

PvuII - CC (N=51, 154.0±14.0), CT (N=117, 154.9±15.8), TT (N=59, 155.2±13.2) p=0.9129;

AluI - AA (N=27 153.4±14.6), AG (N=99, 155.4±14.4), GG (N=101, 154.5±15.3) p=0.8008;

RasI - AG (N=23, 150.4±15.2), GG (N=204, 155.2±14.6) p=0.1392

## Conclusions

In idiopathic scoliosis patients investigated ESR1 and ESR2 gene SNPs showed no association with age at menarche onset.
